# Role of connexins in infantile hemangiomas

**DOI:** 10.3389/fphar.2013.00041

**Published:** 2013-04-16

**Authors:** Katja Blanke, Ingo Dähnert, Aida Salameh

**Affiliations:** Department of Pediatric Cardiology, Heart Center Leipzig, University of LeipzigGermany

**Keywords:** blood vessel, angiogenesis, connexins, infantile hemangioma, β-adrenoceptor, propranolol

## Abstract

The circulatory system is one of the first systems that develops during embryogenesis. Angiogenesis describes the formation of blood vessels as a part of the circulatory system and is essential for organ growth in embryogenesis as well as repair in adulthood. A dysregulation of vessel growth contributes to the pathogenesis of many disorders. Thus, an imbalance between pro- and antiangiogenic factors could be observed in infantile hemangioma (IH). IH is the most common benign tumor during infancy, which appears during the first month of life. These vascular tumors are characterized by rapid proliferation and subsequently slower involution. Most IHs regress spontaneously, but in some cases they cause disfigurement and systemic complications, which requires immediate treatment. Recently, a therapeutic effect of propranolol on IH has been demonstrated. Hence, this non-selective β-blocker became the first-line therapy for IH. Over the last years, our understanding of the underlying mechanisms of IH has been improved and possible mechanisms of action of propranolol in IH have postulated. Previous studies revealed that gap junction proteins, the connexins (Cx), might also play a role in the pathogenesis of IH. Therefore, affecting gap junctional intercellular communication is suggested as a novel therapeutic target of propranolol in IH. In this review we summarize the current knowledge of the molecular processes, leading to IH and provide new insights of how Cxs might be involved in the development of these vascular tumors.

The circulatory system is one of the first systems that develops during embryogenesis. For the development of other organs a sufficient supply with oxygen and nutrients is required, which is mediated through blood vessels as a part of the circulatory system. The formation of blood vessels plays a major role in growth and development of organs in the embryo as well as wound healing and organ regeneration in adulthood (Carmeliet, 2005). The establishment of the circulatory system is a two step process. During vasculogenesis endothelial precursor cells form a primitive vascular network. This primitive network expands during angiogenesis by sprouting and growth of pre-existing vessels, thereby forming new blood vessels. As a result a highly organized vascular network is formed, consisting of arteries, veins, and capillaries (Eichmann et al., 2005). In view of the crucial role of angiogenesis in life, it is not surprising that this process underlies accurate regulatory mechanisms, requiring a finely tuned balance between pro- and antiangiogenic factors (Conway et al., 2001). An imbalance of these mediators and thus, a dysregulation of vessel growth are associated with the pathogenesis of many disorders, especially tumorigenesis (Carmeliet and Jain, 2000; Carmeliet, 2003). Folkman (1971) proposed for the first time the critical role of angiogenesis in tumor growth and metastasis. While angiogenesis is essential for the formation of new blood vessels in the embryogenesis, most blood vessels remain quiescent in adulthood. However, in tumorigenesis there is a shift in favor of proangiogenic factors, which induce angiogenesis and as a result the formation of new blood vessels. The underlying mechanisms of how angiogenesis promotes tumor progression and metastasis are not completely understood. In the past, more and more research focused on the influence of gap junctional coupling during tumorigenesis (Naus et al., 1991; Jamieson et al., 1998; Yamasaki et al., 1999; Saunders et al., 2001; Zhang et al., 2003).

Intercellular communication within the vasculature is essential for vessel formation and the maintenance of normal vascular function. In the vascular system a direct cell-to-cell communication is ensured by transmembrane channels, known as gap junctions. Gap junction channels, linking the cytoplasm of neighboring cells, allow an electrical coupling as well as a metabolic coupling *via* exchange of metabolites, ions, and other messenger molecules up to a molecular mass of 1 kDa. A gap junction channel consists of two hemichannels (connexons), whereby each neighboring cell contributes one hemichannel. Each connexon is composed of six gap junction proteins, called connexins (Cx) (Dhein et al., 2002). In mammals, at least 21 connexin isoforms have been characterized (Söhl and Willecke, 2004). All Cxs are comprised of four transmembrane-spanning domains, two extracellular domains, and a cytoplasmatic amino- and carboxy-terminal region. In the vascular wall four Cxs have been found: Cx37, Cx40, Cx43, and Cx45 (Hill et al., 2001; Isakson et al., 2006, 2008). In most cases, Cx45 is expressed only by smooth muscle cells (Krüger et al., 2000; Li and Simard, 2001; Rummery et al., 2002). In contrast, Cx37, Cx40, and Cx43 have been detected in both, smooth muscle and endothelial cells, while Cx37 and Cx40 are predominantly expressed by endothelial cells and Cx43 is the major connexin isoform in smooth muscle cells (Little et al., 1995; Gabriels and Paul, 1998; van Kempen and Jongsma, 1999; Severs et al., 2001; Haefliger et al., 2004). As Cxs are expressed by endothelial cells as well as smooth muscle cells heterocellular coupling may occur between endothelial cells and smooth muscle cells (myoendothelial junctions). In addition, homocellular coupling between endothelial cells or smooth muscle cells, respectively, does also occur (De Wit, 2004). There is increasing evidence, suggesting a role of vascular gap junctions in the conduction of vasomotor response, the regulation of vascular cell proliferation and migration as well as vascular cell growth, differentiation, and development (Coutinho et al., 2003; Liao et al., 2007; Chadjichristos et al., 2008). However, numerous diseases with vascular abnormalities exhibit an alteration in expression and/or distribution of these Cxs (Laird, 2006; Brisset et al., 2009; Figueroa and Duling, 2009; Johnstone et al., 2009). Previous *in vitro* and *in vivo* studies indicated an influence of gap junctions in tumorigenesis, usually demonstrating a decrease in connexin expression in several neoplastic cells (Czyz, 2008). In addition to vascular abnormalities, mutations in a number of genes, encoding different Cxs, are associated with various further disorders (Kar et al., 2012). For example, mutations in the Cx26 gene (*GJB2*) cause genetic deafness (Martínez et al., 2009). Moreover, mutations in the Cx32 gene (*GJB1*) are involved in the pathogenesis of X-linked Charcot–Marie–Tooth neuropathy (Scherer and Kleopa, 2012), while mutations in the gene encoding Cx43 (*GJA1*) result in oculodentodigital dysplasia (Paznekas et al., 2009). Cxs are also known to play an important role in the heart and previous studies showed several cardiac malformations caused by mutations in the cardiac Cx43 and Cx40 gene (*GJA1* and *GJA5*, respectively) (Delmar and Makita, 2012).

Vascular anomalies are classified into two categories based on their clinico-pathophysiologic behavior and endothelial cell characteristics: vascular malformations and hemangiomas (Mulliken and Glowacki, 1982). While vascular malformations describe structural abnormalities in vessels with normal endothelial turnover, hemangiomas represent vascular tumors, arising as a result of rapid growth of endothelial cells (Bruckner and Frieden, 2003). The most common benign vascular tumor during infancy is the infantile hemangioma (IH).

In this review we summarize the current knowledge of the pathogenesis of IHs and suggest a role of Cxs in the development of these vascular tumors.

Most hemangiomas are not present at birth, but appear during the first month of life with an incidence of 5–10% of all infants, and up to 30% of premature babies, especially those with a birth weight less than 1500 g (Drolet et al., 1999, 2008; Greenberger and Bischoff, 2011). Additionally, there is a higher risk in female than in male infants and it is often seen in Caucasian children (Hemangioma Investigator Group et al., 2007). There are several types of hemangioma. The capillary hemangioma is the most common form of hemangioma, characterized by a closely packed aggregation of small capillaries, separated by thin, connective tissue (Mentzel et al., 1994). The capillaries are normal in size and diameter, but high in number. Another type of hemangioma is the cavernous hemangioma, composing of enlarged, dilated blood vessels with blood-filled cavities between them. A compound hemangioma exists when there is a mix of the capillary and cavernous form. IHs may occur throughout the body, including skin, muscle, bone, and internal organs. Mostly, they are found on the skin of the head and neck area (60%). They can also emerge anywhere else on the skin surface (superficial hemangiomas) like the trunk (25%), limbs (15%) as well as under the skin (deep hemangiomas), and rarely in organs like liver, intestine, lung, or brain (Finn et al., 1983). At first, IHs usually appear as small scratch or bruise red bump, which is why they are also called “strawberry marks”. Depending on the depth of tissue involvement, the hemangiomas display a bright red (outer layers of the skin), crimson, purple, bluish, or normal skin (deep under the skin surface) color. There also exist mixed hemangiomas, combining clinical features of superficial and deep hemangiomas (Chang et al., 2008). The size of IHs may also vary, ranging from a few millimeters to several centimeters in diameter (Drolet et al., 1999). In addition to size there are differences in the shape of hemangiomas. Most tumors are circumscribed and exhibit a round or oval shape, but in some cases they may follow the shape of the affected region. Although, the majority of IHs are solitary and localized, some hemangiomas may be diffuse and segmental, covering a broad range of the cutaneous surface. Hemangiomas, which cannot clearly be classified as localized or segmental hemangiomas, often referred as intermediate hemangiomas (Chiller et al., 2002). The IH displays a characteristic life cycle, consisting of an early proliferation phase and subsequently involution. A few weeks from birth the tumor starts to grow rapidly. The duration of the proliferation phase varies between different hemangioma subtypes, while superficial hemangiomas grow earlier in infancy and faster than deep hemangiomas. However, most growth occurs during the first 4–6 month of life (Hochman et al., 2011). After the proliferation phase, which may last up to 12 month, the tumor undergoes involution and regression. This can take as long as 5–10 years (Phung et al., 2005). In this period the involved skin is blanching and the tumor is shrinking and softening. In 90% of cases IHs regress spontaneously over the years and no specific treatment is required (Margileth and Museles, 1965; Neri et al., 2012). However, hemangiomas may cause disfigurement due to scar tissue or atrophic, wrinkled, telangiectatic skin (Wirth and Lowitt, 1998). In some cases, IHs lead to serious complications, affecting breathing, vision, eating, or hearing, some become life-threatening and require immediate treatment. The management of hemangiomas is non-uniform due to their heterogeneity and depends on several factors like the location and size of the tumor, the depth of the affected region, the age of the patient as well as the occurring complications (Maguiness and Frieden, 2012). Therefore, the treatment of IHs almost follows on a case-by-case basis. Besides surgical management like excision of the hemangioma or laser treatment, children often received a medical therapy (Adams, 2001). Since 1960s corticosteroids have been the standard treatment for IH (Cohen and Wang, 1972). Some infants with life-threatening hemangiomas failed to respond to corticosteroids. In that case the administration of interferon-alpha seemed to be successful in treating of hemangiomas (Ricketts et al., 1994; Chang et al., 1997; Tamayo et al., 1997; Azzopardi and Wright, 2012). Both, corticosteroid and interferon-alpha management are associated with a number of side effects and therefore, the interest in alternate therapies has increased recently (Barlow et al., 1998; Boon et al., 1999). In the last years, advances in the treatment of IHs have occurred. As part of this, a new therapeutic strategy has been added, using propranolol. The therapeutic effect of propranolol was first described by Léauté-Labrèze et al. (2008). They treated a child, suffering from a capillary hemangioma, with steroids. Nevertheless, the hemangioma still grew and simultaneously an obstructive hypertrophic myocardiopathy developed. Therefore, they administered propranolol and observed an involution of the hemangioma. Numerous other studies followed, confirming this inhibitory effect of propranolol on the growth of hemangiomas and thus, this non-selective β-blocker became the first-line therapy for IH (Holmes et al., 2011; Starkey and Shahidullah, 2011). Until today, the effect of propranolol on IH is not completely understood, but with improving knowledge of the pathogenesis of IH several possible mechanisms of action of the β-blocker arised (Storch and Hoeger, 2010). It is believed that propranolol leads to (1) suppression of angiogenesis (2) vasoconstriction of the capillaries, and (3) induction of apoptosis.

Propranolol is a non-selective β-blocker, inhibiting β_1_- and β_2_-adrenoceptors, and has no partial agonistic effect. In the vasculature β_2_-adrenoceptor is the most abundant β-adrenoceptor, which is expressed by a number of cell types, including endothelial cells (Guimarã, 2001; Iaccarino et al., 2005). β-adrenoceptors are a class of G_s_-protein-coupled receptors. Binding of catecholamines on the receptors results in stimulation of the sympathetic nervous system, which plays a major role in regulation of the vascular system. Once the β-adrenoceptors are activated by catecholamines, a series of signaling pathways are initiated. This includes the stimulation of the adenylyl cyclase, which converts adenosine triphosphate (ATP) into cyclic adenosine monophosphate (cAMP). The second messenger cAMP in turn activates the cAMP-dependent protein kinase A (PKA), which phosphorylates numerous intracellular target proteins, involved in the control of cell proliferation, differentiation, and migration. A recent study revealed the important role of β-adrenoceptor stimulation in endothelial cells as a major contributor to the initiation of IH (Mayer et al., 2012). During the characteristic proliferating phase the hemangioma is composed of a highly packed mass of rapidly dividing endothelial cells with increased mitotic rates. Furthermore, pericytes around endothelial cells, and several cell types like mast cells, and myeloid cells in the interstitium of the tumor have been identified. At the involuted phase tumor growth has stopped and an increase in the number of mast cells has been observed. On cellular level these two phases can be separated by specific markers. In the proliferating hemangioma there is an increase in the expression of proangiogenic factors like vascular endothelial growth factor (VEGF) as well as basic fibroblast growth factor (bFGF), platelet-derived growth factor (PDGF), type IV collagenase, insulin-like growth factor 2 (IGF-2), proliferating cell nuclear antigen (PCNA), the integrins α5β3 as well as α5β1, hypoxia-inducible factor (HIF)-1α, and the matrix metalloproteinases (MMP)-2 and MMP-9 (Takahashi et al., 1994; Chang et al., 1999; Ritter et al., 2002; Kleinman et al., 2007; Zhong et al., 2009). In contrast, the involution phase is characterized by a reduced expression of VEGF, bFGF, and IGF-2 as well as an increased expression of the tissue inhibitor of metalloproteinases (TIMP)-1 (Takahashi et al., 1994; Chang et al., 1999; Przewratil et al., 2009, 2010; Zhong et al., 2009). Furthermore, there is an increase in apoptosis, inducing the regression of hemangiomas (Razon et al., 1998).

Vascular endothelial growth factor is a major regulator of vasculogenesis and angiogenesis and was first described as a vascular permeability factor released by tumor cells (Senger et al., 1983). With regard to IH, VEGF seems to play a key role in the pathogenesis of this vascular tumor. VEGF accelerates tumor growth *via* different mechanisms, comprising stimulation of proliferation as well as migration, increased vascular permeability, and inhibition of apoptosis (Harris et al., 2002). It is described that catecholamine-mediated β-adrenoceptor stimulation induces a release and enhanced expression of VEGF in numerous cell types like cancerous cells (Greenberger and Bischoff, 2011; Ji et al., 2013). Furthermore, it has been shown that this is under the control of the cAMP/PKA pathway, activating Src tyrosine kinases and consequently, the extracellular signal-regulated kinase (ERK)/mitogen-activated protein kinase (MAPK) signaling cascade, which in fact results in an enhanced VEGF expression (Eliceiri et al., 1999; Pagès et al., 2000). VEGF exerts its effect, especially *via* a paracrine pathway, by binding to its tyrosine kinase receptor, mostly expressed on endothelial cells. There are several lines of evidence, suggesting a predominant role of the binding of VEGF-A to VEGFR-2 to stimulate angiogenesis (Kieran et al., 2012). The ligand–receptor-interaction again triggers a cascade of signals, including the ERK/MAPK signaling pathway, which results in the phosphorylation of nuclear transcriptional factors and induction of the expression of several genes, responsible for the proliferation of vascular endothelial cells (D’Angelo et al., 1997; Storch and Hoeger, 2010). Therefore, the proangiogenic phenotype is sustained. In a similar fashion, the expression of bFGF is modulated by β-adrenoceptor. Thus, a blockade of the β-adrenoceptor-mediated up-regulation of VEGF and bFGF and hence, an inhibition of angiogenesis by propranolol seem to be important in the management of IH (Ji et al., 2013). Beside β-adrenoceptor stimulation, VEGF expression is also increased by hypoxia, a powerful inducer of vasculogenesis and angiogenesis (Sakurai and Kudo, 2011). Like many other tumors IH develops a hypoxic microenvironment, which becomes obvious by increased expression and stabilization of HIF-1α during the proliferation phase of the tumor. Moreover, previous studies demonstrated a link between β-adrenoceptors and HIF-1α in several cancer cells, indicating an up-regulation of HIF-1α by catecholamine-mediated β-adrenoceptor stimulation also under normoxic conditions (Chim et al., 2012). Recently, it has been shown that the Src tyrosine kinases are involved in the hypoxia-induced VEGF expression by transactivating the epidermal growth factor receptor (EGFR) tyrosine kinase, which in turn stimulates the Akt and ERK1/2 pathways (Hu et al., 2010). These data suggest a role for propranolol in IH *via* suppression of the HIF-1α- mediated VEGF expression. Collectively, one possible mechanism of action of propranolol on IH might be the reduction of the expression of proangiogenic factors like VEGF and consequently, inhibition of angiogenesis. In relation to its antiangiogenic effect in IH, propranolol could also act *via* inhibition of the expression of MMPs, the most prominent proteinase family associated with tumorigenesis. The involvement of MMPs in IH is supported by the finding of elevated concentrations of MMP-2 and MMP-9 in the proliferation phase. Furthermore, it is well described that catecholamine-mediated stimulation of β-adrenoceptors leads to an increased expression of MMP-2 and MMP-9 (Storch and Hoeger, 2010). Based on the role of MMP-2 and MMP-9 in the migration of endothelial cells and tubulogenesis, a reduction of the expression of these proteinases by propranolol also indicates an antiangiogenic effect of the β-blocker. This is in accordance with the growth arrest and shrinking of the tumor after treatment with propranolol.

Another hypothesized effect of propranolol in IH is the induction of vasoconstriction. In this regard, VEGF again seems to be crucial. VEGF is able to increase vascular permeability and mediates vasodilatation, both coupled to the formation of nitric oxide (NO), and therefore contributes to tumor growth. In their study Parenti et al. (1998) revealed the intracellular pathway of the VEGF/NO-induced proliferation of endothelial cells. They demonstrated an increase in cytosolic calcium [apparently by activation of phospholipase C γ (PLC γ)] upon VEGF stimulation, activating the calcium/calmodulin-dependent endothelial nitric oxide synthase (iNOS), which causes the production and release of NO. NO in turn stimulates the guanylyl cyclase/cyclic guanosine monophosphate (cGMP)/protein kinase G (PKG) cascade, resulting in vasodilatation of endothelial cells. Furthermore, cGMP activates the ERK/MAPK signaling pathway, leading to cell proliferation. Catecholamines are also able to mediate vasodilatation by stimulation of β_2_-adrenoceptors (Storch and Hoeger, 2010). Accordingly, another possible mechanism of how propranolol affects IH is the prevention of relaxation of capillary endothelial cells, consequently leading to vasoconstriction. The link between β-adrenoceptors, VEGF expression, and NO formation points out that there is a complex interaction of multiple signaling cascades, causing vasodilatation. The vasoconstrictive effect of propranolol on hemangiomas is emphasized by the blanching and softening of the tumor after propranolol application.

The third mechanism of action of propranolol in IH represents the induction of apoptosis. Previous studies demonstrated a role of β-adrenoceptor signaling in cell survival, implicating the β-adrenoceptor-induced transactivation of EGFR *via* the Src tyrosine kinase as a critical step. This transactivation stimulates a number of antiapoptotic signaling cascades, including the MAPK cascade, the phosphatidylinositol 3-kinase (PI3K)/Akt pathway as well as the nuclear factor κB (NFκB) cascade (Zhang et al., 2009). As a result, cell proliferation is stimulated, expression of antiapoptotic proteins is induced, and the caspase cascade is inhibited. Hence, propranolol could induce apoptosis by preventing the β-adrenoceptor-mediated cell survival.

Among the above mentioned mechanisms, the success of propranolol therapy in IH might also be attributable in affecting intercellular communication. In the past, it has been described that a reduction in intercellular communication is associated with increased susceptibility of cells to neoplastic transformation (Geletu et al., 2012). As gap junction channels conduct growth-regulating signals from cell-to-cell, an impaired gap junctional coupling results in uncontrolled cell growth and therefore, leading to tumor promotion. Furthermore, studies, investigating the involvement of gap junction intercellular communication (GJIC) in carcinogenesis, confirmed a major role of connexin expression and localization in the control of cell growth (Yamasaki et al., 1999). Many reports show a down-regulation of connexin expression in numerous neoplastic cell lines and primary tumors (Czyz, 2008). Interestingly, a previous study revealed a role of Cxs in the pathogenesis of hemangiomas (Simon and McWhorter, 2002). They found that mice, lacking both Cx37 and Cx40 die perinatally with abnormalities in the vascular system. These vascular anomalies include hemorrhages in skin, testis, intestine, stomach, and lung, accompanied by dilated blood vessels and congestion in the affected tissue. Furthermore, they observed abnormal vascular channels, coalescing into a cavernous blood pool. These vascular abnormalities were only detected when both Cx37 and Cx40 are absent. In contrast, mice lacking either Cx37 or Cx40 are viable and do not exhibit such severe vascular phenotypes. Thus, the authors suggested an overlapping function of both Cxs in the development and maintenance of the vasculature in mouse. In a follow-up study the authors could confirm the dependency of Cx37 and Cx40 on each other in the vascular endothelium, demonstrating that specific ablation of either endothelial Cx37 or Cx40 results not only in an elimination of the target connexin, but also in a substantial decrease of the non-ablated connexin (Simon and McWorther, 2003). Moreover, they revealed a reduced interendothelial dye-transfer in aorta of mice, lacking Cx37 or Cx40, respectively. The effect of Cx40 ablation on dye-transfer was more pronounced than ablation of Cx37 and was age-dependent. Elimination of both Cxs in embryonic aortas completely abolished dye-transfer in these mice. Thus, expression of Cx37 and Cx40 could be crucial for effective endothelial coupling by forming heteromeric gap-junction channels, which in turn are required for normal development and functional maintenance of the mouse vascular endothelium. This is in accordance with other reports, indicating a coexpression of Cx37 and Cx40 in endothelial cells (Yeh et al., 1998; Ko et al., 1999; Simon and McWhorter, 2002). Other studies also demonstrated the important role of Cx37 and Cx40 in the vascular system, implicating a decrease in intercellular communication and consequently, an impairment of angiogenesis after knock-down of these Cxs (Gärtner et al., 2012). A lot of research has been carried out to elucidate the underlying mechanisms of the regulation of connexin expression, most of them focused on Cx43. The β-adrenoceptor-mediated signaling pathway has been identified as a major modulator of the Cx43 expression. It has been postulated that β-adrenoceptor stimulation leads to a PKA-dependent activation of the three MAPK p38, c-Jun N-terminal kinase (JNK), and ERK1/2, resulting in a translocation of transcriptional factors like activator protein 1 (AP1) and cAMP response element-binding protein (CREB) into the nucleus, which finally bind to the Cx43 promoter and thus, inducing connexin expression (Salameh et al., 2009). Furthermore, β-adrenoceptor stimulation induces elevated concentration of intracellular calcium, which in turn activates the calcineurin pathway. The protein phosphatase calcineurin dephosphorylates the transcriptional factor nuclear factor of activated T cells (NFAT), which also translocates into the nucleus and induce the expression of Cx43 (Salameh et al., 2009). Although, less data are available of the regulation of Cx37 and Cx40, these observations indicate an involvement of β-adrenoceptor signaling in the regulation of their expression. For example, this is supported by the study of Dupays et al. (2003), detecting multiple binding sites for AP 1 in the Cx40 gene and hence, suggesting an important role of this transcription factor also in the expression of Cx40. In this regard, a further mechanism of action of propranolol in IH might affect connexin expression. Suprisingly, a recent study demonstrated an increased Cx43 protein expression in rat cardiomyocytes after metoprolol application (Salameh et al., 2010). Since these findings do not fit into the classical scheme of action of the β_1_-selective adrenoceptor blocker, it is postulated that metoprolol exerts this Cx43-elevating effect *via* a β_1_-adrenoceptor independent mechanism, possibly by inhibition of Cx43 degradation. This so-called ancillary effect of β-blockers is well known and could also be observed for propranolol. Marano et al. (2002) revealed an antihypertrophic effect of propranolol, presumably due to its membrane-stabilizing effect and independently from its β-adrenergic blocking activity. Hence, propranolol may increase connexin expression independent of β-adrenoceptor blockade. Beside the connexin expression, GJIC is regulated by posttranslational modifications of Cxs, especially phosphorylation, ensuring a correct assembly and establishment of a complete functional gap junction channel. The carboxy-terminal region of Cxs contains consensus sequences, which can be phosphorylated by several protein kinases. Depending on the protein kinase, phosphorylating the connexin, and the phosphorylated amino residue (serine, threonine, or tyrosine) of the connexin, an increase or decrease in GJIC may occur (Dhein et al., 2002). Previous data indicated that Src tyrosine kinase is able to phosphorylate Cx43 on tyrosine residue (tyr) 247 and tyr265, resulting in reduced GJIC. In addition, Src initiates multiple signaling cascades, activating a number of protein kinases, which in turn phosphorylate Cx43 (Pahujaa et al., 2007). On the one hand, Src activates the Ras/Raf/MEK/MAPK pathway. MAPK are serine/threonine-selective protein kinases, which can also phosphorylate Cx43 on serine residues and block GJIC (Warn-Cramer et al., 1996). On the other hand, Src stimulates PLC, leading to an increase in cytosolic calcium levels and activation of the calcium-dependent protein kinase C (PKC). Additionally, Src may phosphorylates PKC and thus, directly activates this protein kinase. PKC, a serine/threonine protein kinase, is again able to phosphorylate Cx43 on serine residues, which mostly correlates with a reduction in GJIC (Pahujaa et al., 2007). As described above, Src-mediated signaling cascades play a pivotal role in the pathogenesis of IH *via* inducing the expression of angiogenic factors, causing vasodilatation, and inhibiting of apoptosis. Up to now, the suggested explanations for the therapeutic effect of propranolol, leading to regression of IH, focused on the prevention of these events. However, it is also conceivable that the blockade of the Src-mediated pathways by propranolol suppress connexin phosphorylation, leading to enhanced GJIC. Recently, a study of Johnstone et al. (2012) revealed a MAPK-mediated phosphorylation of Cx43 at the specific serine residues of its C-terminus, promoting vascular smooth muscle cell (VSMC) proliferation, independently of GJIC. It has been shown that in response to PDGF Cx43 become phosphorylated by MAPK, followed by an interaction of the phosphorylated Cx43 with the cell cycle regulator cyclin E and its associated kinase cyclin-dependent kinase (CDK)-2 at the cell membrane. Subsequently, the complex of phosphorylated Cx43, cyclin E and CDK-2 internalizes and activates downstream targets of cyclin E and CDK-2, like retinoblastoma (Rb) protein, which in turn stimulates VSMC proliferation. They could also observed that this MAPK-mediated Cx43 phosphorylation is crucial in regulation of neointima formation *in vivo*. Due to the fact that PDGF is elevated in the proliferation phase of IH, the PDGF-induced proliferation of VSMC through mechanisms, involving MAPK-mediated Cx43 phosphorylation and complex formation of phosphorylated Cx43 with cyclin E and CDK-2, could also play an important role in the pathogenesis of IH. As described above, β-adrenoceptor stimulation results in activation of MAPK, thereby enhancing the phosphorylation of Cx43, which could be suppressed by propranolol (Salameh et al., 2009). Furthermore, Ji et al. (2013) demonstrated that β-adrenoceptor antagonists interact with cell cycle regulators, for example phospho-Rb, and thus, inhibit cell proliferation. Therefore, it could be suggested that propranolol prevent the β-adrenoceptor-mediated activation of the MAPK cascade and thus, the phosphorylation of Cx43. Hence, no interaction of Cx43 with cell cycle regulators occur and promotion of cell proliferation is avoided, which finally could lead to regression of the IH.

Until today, the role of Cxs in IH is not fully understood and further experiments are required to clarify the involvement of Cxs in the development of IH. As described by Gärtner et al. (2012) knock-down of Cx37, Cx40, and Cx43, respectively, *via* siRNA interference in 3D Matrigel culture of human umbilical vein endothelial cells (HUVEC) resulted in an impairment of capillary network formation. It would be interesting to determine the capillary formation in an angiogenesis assay of endothelial cells of mice, lacking both Cx37 and Cx40, which showed hemorrhages in different organs (Simon and McWhorter, 2002). Based on the findings of Simon and McWorther (2003) that elimination of Cx37 and Cx40 lead to a complete loss of endothelial dye-transfer in embryonic aortas of mice, Patch clamp analysis could provide further insights into the functionality of gap junction channels in the aortic wall of these mice by investigation of the electrical gap junctional coupling. In addition, expression and activity of key regulators of the connexin expression, for example Src kinase, MAPK, transcription factors, as well as downstream targets of Cxs, involved in cell cycle regulation and cell proliferation, should be studied in hemangioma tissue of these mice. Among animal models, an important starting point to reveal a role of Cxs in IH might be to determine the expression and localization of these gap junction proteins in biopsies of infants with IH. In a next step, the above mentioned molecular pathways, possibly involved in IH, could be examined in human hemangioma tissue.

In summary, there are some explanations for the therapeutic effect of propranolol on IH, including the reduction of the expression of angiogenic factors, stimulation of vasoconstriction, and triggering of apoptosis. Although, the underlying mechanisms are not completely understood, there is evidence of a pivotal role of β-adrenoceptor stimulation, leading to the activation of a number of signaling pathways. Furthermore, these pathways are also involved in the regulation of Cxs. In this regard, another mechanism of action of propranolol has been postulated *via* enhancement of GJIC and inhibition of cell proliferation by affecting connexin expression and phosphorylation. All these possible mechanisms of action of propranolol in IH are illustrated in **Figure 1**.

**FIGURE 1:**
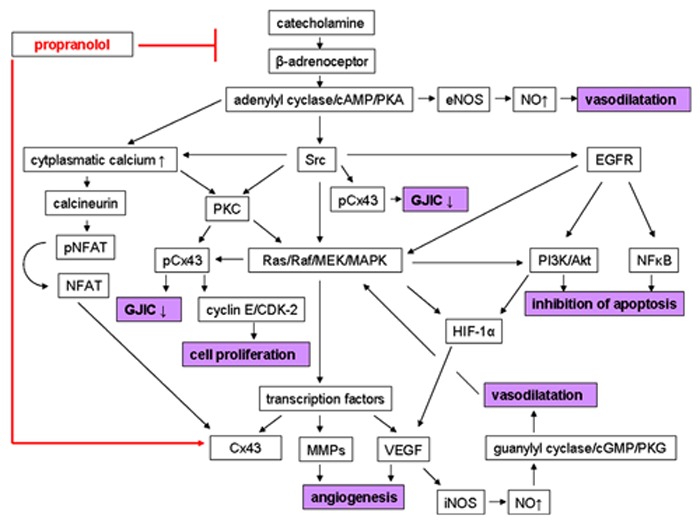
**Possible mechanisms of action of propranolol in infantile hemangiomas (IHs).** In the absence of the β-adrenoceptor blocker a catecholamine-induced stimulation of the β-adrenoceptor activates several signaling pathways, leading to the pathophysiology of IH. This includes signal cascades, causing (1) angiogenesis *via* induction of the expression of proangiogenic factors like VEGF and MMPs (2) vasodilatation *via* formation and release of NO, (3) inhibition of apoptosis, and (4) reduction of GJIC and stimulation of cell proliferation, respectively, *via* affecting connexin expression and phosphorylation. In contrast, propranolol treatment results in inhibition of angiogenesis, stimulation of vasoconstriction, triggering of apoptosis, increase of GJIC, and prevention of cell proliferation *via* blockade of these β-adrenoceptor-mediated pathways, and thus leading to regression of IHs. Furthermore, propranolol could affect connexin expression independently from its β-adrenergic blocking activity (see text for details). Arrows indicate activation; ↑ indicates up-regulation; ↓ indicates down-regulation; ⊥ indicates inhibition. cAMP, cyclic adenosine monophosphate; CDK-2, cyclin-dependent kinase 2; cGMP, cyclic guanosine monophosphate; Cx43, connexin43; EGFR, epidermal growth factor receptor; eNOS, calcium-independent endothelial nitric oxide synthase; GJIC, gap junctional intercellular communication; HIF-1α, hypoxia-inducible factor 1; iNOS, calcium-dependent endothelial nitric oxide synthase; MAPK, mitogen-activated protein kinases; MMP, matrix metalloproteinase; NFAT, nuclear factor of activated T cells; NFκB, nuclear factor κB; NO, nitric oxide; pCx43, phosphorylated connexin43; PI3K, phosphatidylinositol 3-kinase; PKA, protein kinase A; PKC, protein kinase C; PKG, protein kinase G; pNFAT, phosphorylated nuclear factor of activated T cells; VEGF, vascular endothelial growth factor.

## CONCLUSION

Over the last years, knowledge of the molecular mechanisms of IH has improved, clarifying that several signaling cascades are involved in the progression of these vascular tumors and that there is a cross-talk between them. Since propranolol has been identified as a new therapeutic option in treating IH, various studies focused on the underlying mechanisms of how propranolol affects hemangiomas. Until today, propranolol has become the first-line therapy in IH and some possible mechanisms of action of propranolol in IH have been postulated, implicating Cxs as a novel therapeutic target of propranolol in IH. The challenge for the future will be to elucidate the cellular and molecular basis and pathways of IH in greater detail to completely understand the mechanism of action of propranolol and finally, to optimize therapy for infants with IH.

## Conflict of Interest Statement

The authors declare that the research was conducted in the absence of any commercial or financial relationships that could be construed as a potential conflict of interest.
